# Enhancing Psychological Resilience: Examining the Impact of Managerial Support on Mental Health Outcomes for Saudi Ambulance Personnel

**DOI:** 10.3390/healthcare11091277

**Published:** 2023-04-29

**Authors:** Ahmed M. Al-Wathinani, Mohannad A. Almusallam, Nawaf A. Albaqami, Mohammed Aljuaid, Abdullah A. Alghamdi, Mohammad A. Alhallaf, Krzysztof Goniewicz

**Affiliations:** 1 Department of Emergency Medical Services, Prince Sultan bin Abdulaziz College for Emergency Medical Services, King Saud University, Riyadh 11451, Saudi Arabia; 2 National Guard Health Affairs, King Abdulaziz Medical City, Dirab Primary Health Center Riyadh, Riyadh 14972, Saudi Arabia; almuslemmu@mngha.med.sa; 3 Department of Health Administration, College of Business Administration, King Saud University, Riyadh 11451, Saudi Arabia; 4 Department of Security Studies, Polish Air Force University, 08-521 Dęblin, Poland; k.goniewicz@law.mil.pl

**Keywords:** emergency medical services, ambulance personnel, paramedics, mental health, behavior, critical care, medical risk factor, psychological and psychosocial

## Abstract

Ambulance personnel are among the groups with high mental health risks. This study aims to investigate the role of managerial support in determining the mental well-being of ambulance personnel, a group at high risk for mental health issues. A descriptive, cross-sectional survey design was conducted in Riyadh, Saudi Arabia, in February 2022, involving a convenience sample of 354 ambulance personnel. An online survey was distributed via social media platforms. Manager behavior and mental well-being were assessed using the Manager Behavior Questionnaire (MBQ) and the Short Warwick-Edinburgh Mental Well-being Scale (SWEMWBS). The participants represented nearly equal-sized groups from different agencies, with 50.3% residing in the Riyadh Region and 67.5% aged between 25 and 34. The mean score for manager behavior was 2.92 ± 1.124, while the mental well-being scale’s mean score was 3.398 ± 0.8219. Variance analyses revealed statistically significant differences in manager behavior concerning gender, age, residence, and years of experience (*p* < 0.05), as well as in the mental well-being of ambulance personnel. Generalized linear regression analysis demonstrated a statistically significant relationship between manager behavior and mental well-being (*p* < 0.01). Focusing on improving organizational management behaviors is a promising strategy for enhancing mental health interventions among ambulance personnel. Further research is recommended to monitor the mental health of these professionals and develop evidence-based interventions to support their well-being.

## 1. Introduction

The World Health Organization highlights the importance of mental health as part of overall well-being, encompassing not just the absence of mental disorders or disabilities, but also the ability to realize one’s potential, cope with life’s stresses, work productively, and contribute to the community [1]. Workplace stress is a critical issue, impacting both employees’ health and well-being and an organization’s productivity. High-stress occupations, such as emergency medical services (EMS), face an elevated risk of depression and anxiety [2].

Emergency health professionals’ performance and output are significantly affected by stress, resulting in high emotional costs [3]. Prehospital settings, in particular, subject EMS workers to constant emotional strain. Stress levels among these workers can range from 6% to 80% [4,5]. Unpredictable environments, as well as daily and cumulative trauma contribute to mental distress and psychological injury among ambulance personnel, who encompass individuals employed by emergency service provider agencies involved in prehospital or interhospital transport of patients requiring emergency care or life support services [6]. Because of their workload, ambulance personnel often report increased psychiatric and psychological distress symptoms, including post-traumatic stress disorder (PTSD), depression, anxiety, and general psychological distress [7].

Paramedics, as frontline healthcare professionals, are frequently confronted with a myriad of highly stressful situations that demand effective coping mechanisms for dealing with unforeseen and non-specific threats [8]. In countries such as South Africa, EMS reports suggest that employees experience critical incidents at a higher frequency compared with their counterparts in more developed nations. [9]. Similarly, Saudi paramedics have been found to experience elevated levels of depression, PTSD, sleeplessness, exhaustion, and diminished physical functioning when compared with their Australian counterparts [10].

Despite the apparent need for psychological support, the Psychological Support Unit (PSU) of the Saudi Red Crescent Authority (SRCA) reveals that only a small percentage of cases reported to the PSU for psychological assistance and management are from SRCA workers [11]. This gap in accessing mental health services may be attributed to various factors, such as stigma surrounding mental health, lack of awareness of available resources, or insufficient organizational support.

EMS providers confront a wide array of challenges that can impede their effectiveness in delivering critical care. To bolster their efficiency, it is vital to thoroughly assess the scope of these difficulties, eradicate barriers, and offer comprehensive education and training programs [12]. Interventions aimed at enhancing mental health and well-being in industries with higher risks, such as EMS, have traditionally focused on individual-level factors. However, there is a growing interest in organizational-level interventions that recognize the significant role of managerial behavior in shaping a team’s success, morale, employee turnover, and overall workplace culture [13].

Organizational-level factors play a critical role in shaping the work environment, with the potential to influence mental health outcomes and job satisfaction among employees. In the EMS industry, it is essential to examine the impact of managerial practices, communication styles, and resource allocation on stress levels and well-being. By fostering a supportive atmosphere that emphasizes trust and open communication, EMS organizations can enhance employee well-being, leading to improved performance, retention, and overall service quality. Consequently, exploring the relationship between managerial support and the mental health of ambulance personnel is crucial for devising effective strategies that contribute to the improvement of emergency health systems.

The primary objective of this study is to examine the importance of managerial support in determining the mental well-being of ambulance personnel in Saudi Arabia, considering the heightened mental health risks they encounter. Gaining insight into the influence of managerial support on mental well-being is essential for devising effective interventions and strategies aimed at improving the overall mental health of these professionals. By highlighting the connection between managerial behavior and mental well-being, our research offers valuable guidance for policymakers and organizations in developing a supportive work environment that fosters mental health among frontline workers. Ultimately, this will enhance the quality of emergency medical services and contribute to the well-being of the communities they serve.

## 2. Materials and Methods

### 2.1. Sample and Procedures

The study, conducted in Riyadh, Saudi Arabia, in February 2022, employed a robust descriptive cross-sectional survey design to investigate the relationship between managerial support and mental health among ambulance personnel. The target population consisted of various categories of ambulance personnel, including call takers, ambulance dispatchers, and front-line EMS personnel such as emergency medical technicians, paramedics, and intensive care paramedics, currently employed by the Saudi Red Crescent Authority (SRCA), government, and private health sectors in Saudi Arabia. Administrators, volunteers, and trainees were excluded from the study.

Demographic characteristics encompassed gender, age, nationality, region of residence, current employer (governmental or private sector), and years of experience. Additionally, the participants were categorized based on their skill levels, such as emergency medical technicians, paramedics, and intensive care paramedics. The employer region was categorized into five main regions—central, northern, western, southern, and eastern—that encompass all 13 administrative areas of Saudi Arabia.

We aimed to identify any notable differences between governmental and private sector organizations, particularly concerning the impact and behaviors of management. This allowed for a more comprehensive understanding of how organizational context may influence the relationship between managerial support and mental health among ambulance personnel.

### 2.2. Measures

Data collection utilized a modified questionnaire adapted from a previous open-access study that examined the importance of different managerial support factors in determining the mental health of ambulance personnel in Australia [12]. The modifications were made to better suit the cultural context and organizational structure of ambulance services in Saudi Arabia. The questionnaire integrated the Short Warwick–Edinburgh Mental Well-being Scale (SWEMWBS) to assess mental well-being. SWEMWBS has been validated in both community and clinical samples [13], and primarily focuses on measuring an individual’s mental well-being. The scale consisted of seven positively phrased items, assessing well-being over the last two weeks on a five-point Likert scale. Total scores ranged from 7–35, with higher scores indicating greater overall psychological well-being.

Managerial behavior was assessed using a reliable and validated nine-item scale, known as the Managerial Behavior Questionnaire (MBQ). This questionnaire gauges employees’ direct experiences of various aspects of their manager’s behavior (Cronbach’s alpha = 0.95) [12] and is designed to measure the perceived supportiveness of managerial behaviors. The positively worded items were evaluated on a five-point Likert frequency scale and summed to give a total score ranging from 9–45. Lower scores indicated less supportive, critical, and distant managers, while higher scores represented more frequent supportive managerial behaviors, such as providing encouragement and resources.

To ensure the anonymity of respondents, no personally identifiable information was collected during the survey process. All responses were coded using unique identifiers, and the data were stored securely with access limited to the research team.

The survey link was disseminated to ambulance personnel through Twitter, WhatsApp, and other social media platforms from February to April 2022.

### 2.3. Statistical Tools

Data analysis was performed using the Statistical Package for the Social Sciences (SPSS v26). Demographics, manager behavior, and mental well-being were assessed through frequency tables, reliability and validity tests, relative importance tests, variance analyses, descriptive statistics, and *t*-tests. Generalized linear regression models were employed to establish the relationship between managerial support and the mental well-being of ambulance personnel.

### 2.4. Ethical Considerations

Institutional review board (IRB) approval was obtained from the King Saud University Research and Ethics Committee (Ref No: KSU-HE-22-060) in February 2022.

## 3. Results

A total of 400 participants completed the online survey. After excluding invalid responses from administrators, volunteers, and trainees (n = 45), the final sample consisted of 354 ambulance personnel. The majority of the participants were male (84.5%), Saudi nationals (98.9%), and aged between 25 and 34 years old (67.5%). Over half of the respondents (59.6%) were single. The predominant educational level among participants was a bachelor’s degree (79.4%), and nearly half (46.9%) had one to five years of work experience in the ambulance field.

The study collected responses from 11 out of the 13 administrative regions in Saudi Arabia, with approximately half of the participants (50.3%) residing in the Riyadh Region. The respondents represented a fairly balanced distribution across agencies, with 37.9% from the SRCA, 36.4% from the governmental health sector, and 25.7% from the private health sector. The regional distribution of employer locations showed varied response rates. The majority of participants worked in the central region (48%), followed by the eastern, western, southern, and northern regions. Detailed characteristics of the participants are presented in Table 1.

### 3.1. Descriptive Statistics and t-Test

Descriptive statistics for the study dimensions (manager behavior and mental well-being) were calculated, including the mean, standard deviation, coefficient of variation, and *t*-test. Table 2 and Table 3 display the descriptive statistics and *t*-test results for the elements of the manager behavior and mental well-being scales, while Appendix A and Appendix B contain Table A1 and Table A2, which present the frequency distribution and percentage of the items for both scales.

The manager behavior scale had a mean of 2.92 ±1.124, indicating that the majority of the sample exhibited neutrality, rather than disagreement, with the elements. In contrast, the mental well-being scale yielded a mean of 3.398 ± 0.8219, signifying that most of the sample agreed with the elements. The coefficient of variation for all dimensions was less than 50, demonstrating low dispersion around the mean. The *t*-test revealed a level of significance for elements (1) and (5) of the manager behavior scale at *p* < 0.05, with an average lower than that of (3); hence, the responses disagreed with these statements. All elements of the mental well-being scale exhibited a level of significance at *p* < 0.05, rendering all statements statistically significant.

### 3.2. Factors Associated with Manager Behavior

The Mann–Whitney test revealed statistically significant differences in manager behavior based on the gender of the ambulance personnel (*p* < 0.05). The Kruskal–Wallis test demonstrated statistically significant differences in manager behavior according to the participants’ age, residence, and experience (*p* < 0.05) (refer to Appendix C, Appendix D, Appendix E and Appendix F: Table A3, Table A4, Table A5 and Table A6).

### 3.3. Factors Associated with Mental Well-Being of Ambulance Personnel

The Kruskal–Wallis test indicated statistically significant differences in the mental well-being of ambulance personnel based on their region of residence (*p* < 0.05). Additional details can be found in Appendix E: Table A5.

### 3.4. Impact of Manager Behavior on Mental Well-Being

A generalized linear regression model (Normal with Identity link) was employed to determine whether the independent variable (manager behavior score) had a statistically significant impact on the dependent variable (mental well-being score).

The normal regression model, based on a normal distribution, linked the independent variables to the predicted value of the dependent variable through the identity link function. The goodness-of-fit results suggested the best model, as illustrated in Table 4. The Omnibus Test showed that the model was significant (*p* < 0.01), as presented in Table 5.

The model parameters and standard errors for each estimate, including the Wald chi-square value, R-square value, and level of significance, were obtained. The manager behavior score significantly influenced the mental well-being score (*p* > 0.01), with an R-square of 0.35 (Table 6). The remaining percentage was either a random error or could be attributed to other independent variables not included in the model.

## 4. Discussion

This study examined the association between managerial support and the mental health of ambulance personnel, finding a statistically significant relationship between managerial support and the mental well-being of ambulance personnel in Saudi Arabia. This finding is consistent with a previous study conducted on ambulance personnel in Australia [14]. Our results identified statistically significant differences in managerial behavior based on gender, age, residence, and years of experience among ambulance personnel, as well as their mental well-being, in relation to their residential region.

One potential challenge faced by ambulance personnel in this study could be the difficulty in feeling relaxed amidst the high-pressure work environment. A possible managerial deficiency that emerged might be the inadequate attention given to the feelings and concerns of these frontline workers. This gap in support could lead to difficulties in effectively communicating work-related stress to their supervisors, leaving ambulance personnel feeling unheard and unsupported. Such communication barriers may contribute to a cumulative negative impact on the mental well-being of ambulance personnel, potentially leading to burnout, reduced job satisfaction, and increased turnover. Therefore, it is essential for organizations to consider the importance of open lines of communication and empathy from management towards their staff. By addressing these managerial shortcomings, organizations may create an environment in which ambulance personnel feel more comfortable discussing their concerns and stressors. In turn, this could enable the development and implementation of targeted support strategies to help alleviate stress, promote a healthy work–life balance, and ultimately enhance the mental well-being and overall performance of these essential frontline workers.

Poor psychological well-being can negatively impact medical professionals’ quality of life, which may, in turn, affect the quality of medical services they provide [15,16]. Identifying and addressing the factors contributing to workplace stress are essential for effectively managing and preventing mental health issues. Previous research has linked stress, depression, and anxiety to the level of engagement in patient care among EMS workers [17,18], with factors such as participants’ age, sex, workload, work duration, and sleep duration being examined.

Psychosocial support plays a crucial role in enabling interventions that help individuals process incidents more effectively, mediate strong emotions, change beliefs, and cope with situations to function more efficiently. Maintaining mental health at the core of operations is critical for enhancing the psychological resilience of ambulance personnel [19], who are constantly exposed to emotional strain. Stress levels among EMS workers can range from 6% to 80% [3,4], with anxiety, depression, and PTSD being associated with the pressures of emergency care and empathy for patients’ needs [3,19]. A study from Riyadh found anxiety disorders in 20.7%, 23.7%, and 7.6% of Saudi Arabia’s emergency department staff, respectively [17].

Mental health is the cornerstone of our collective and individual abilities as humans to think, emote, interact, earn a living, and enjoy life. As such, promoting, protecting, and restoring mental health are essential concerns for individuals, communities, and societies across the globe [1]. Ambulance personnel and their managers, due to the nature of their work, face unpredictable and often high-stress situations that can significantly impact their mental well-being. In recognition of these challenges, the Department of Health [20] has emphasized the importance of a psychosocial approach to support employees, advocating for strategies that complement the employer’s responsibility to safeguard the mental health and well-being of their team [21,22]. This approach involves understanding and addressing the unique mental health needs of ambulance personnel, providing resources for stress management, and fostering a supportive work environment that promotes open communication, resilience, and overall psychological wellness.

This study highlights the crucial role of managerial support in shaping the mental well-being of ambulance personnel in Saudi Arabia. The findings stress the importance of organizations concentrating on enhancing managerial behavior, cultivating open communication channels, and offering resources to tackle the mental health challenges confronted by these frontline workers. By comprehending the relationship between manager behavior and mental well-being, organizations can develop customized interventions and foster a nurturing work environment that bolsters the overall health and resilience of ambulance personnel [22,23]. Incorporating comprehensive mental health support within the organization not only benefits the ambulance personnel themselves, but also results in a positive ripple effect throughout the emergency medical services sector. By addressing the mental well-being of these workers, organizations may improve employee retention, reduce burnout, and enhance overall job satisfaction. This, in turn, may leads to higher-quality emergency medical services, better patient care, and strengthened public health outcomes [24,25,26,27,28]. Ultimately, investing in the mental health and well-being of ambulance personnel is a vital step towards creating a more resilient and effective emergency medical service system that benefits both the workers and the communities they serve.

## 5. Limitations

This his study has several limitations that should be considered when interpreting the results. Firstly, the cross-sectional nature of this study prevents us from establishing a causal relationship between managerial support and the mental well-being of ambulance personnel. Longitudinal studies are needed to determine the directionality of these associations.

Secondly, the study relied on self-reported data from participants, which may introduce bias due to social desirability, recall errors, or misunderstanding of questions. Future research could benefit from incorporating objective measures or observations to validate the findings. Additionally, the study used a convenience sampling method, which may limit the generalizability of the results. The participants may not be representative of the entire population of ambulance personnel in Saudi Arabia. A random sampling approach would provide more generalizable findings.

The survey distribution through social media platforms might also introduce selection bias, as not all ambulance personnel may be active on these platforms or may choose to participate. This may limit the representativeness of the sample.

Some variables were measured using single items, which may not capture the full complexity of the constructs. Future studies should consider using more comprehensive, multi-item measures to assess variables of interest. Finally, the study was conducted in Saudi Arabia, and the findings may not be generalizable to ambulance personnel in other countries with different cultural, organizational, and healthcare contexts.

## 6. Conclusions

This study examined the association between manager support and the mental well-being of currently employed ambulance personnel. The findings highlight the importance of providing appropriate support to ambulance personnel, who face high levels of stress and emotional strain in their daily work. By addressing the role of managerial behavior in the mental well-being of these frontline workers, healthcare organizations can improve the overall quality of emergency medical services and foster a healthier, more resilient workforce.

The study identifies the need for organizational interventions that focus on enhancing managerial support to promote a positive working environment for ambulance personnel. By improving communication, providing resources, and offering encouragement, managers can contribute to the mental well-being of their teams. This, in turn, can lead to better performance, reduced employee turnover, and improved patient care.

Given the high stress and emotional demands associated with ambulance work, it is crucial for healthcare organizations and policymakers to prioritize the mental well-being of these professionals. This study offers valuable insights into the factors associated with the mental health of ambulance personnel, paving the way for future research and the development of evidence-based interventions.

Ultimately, the promotion, protection, and restoration of mental health among ambulance personnel should be a central concern for healthcare organizations and policymakers. By understanding and addressing the factors that influence mental well-being in this high-risk population, we can ensure the ongoing delivery of high-quality emergency medical services and support the health and well-being of those who dedicate their lives to saving others.

## Figures and Tables

**Table 1:** Frequency of demographics (N = 354).

		Frequency	%
Sex	Male	299	84.5
	Female	55	15.5
Age	<25	68	19.2
	25–34 years	239	67.5
	35–44 years	37	10.5
	45–54 years	6	1.7
	55–60 years	3	0.8
	>60	1	0.3
Nationality	Saudi	350	98.9
	Non-Saudi	4	1.1
Residence	Riyadh Region	178	50.3
	Makkah Region	60	16.9
	Madinah Region	15	4.2
	Qasim Region	6	1.7
	Eastern Province	58	16.4
	Asir Region	8	2.3
	Tabuk Region	6	1.7
	Hail Region	3	0.8
	Jazan Region	5	1.4
	Najran Region	9	2.5
	Bahah Region	1	0.3
	Outside Saudi Arabia	2	0.6
	Unknown	3	0.8
Marital Status	Married	138	39.0
	Single	211	59.6
	Divorced	5	1.4
Level of Education	High school or less	3	0.8
	Diploma	29	8.2
	Bachelor	281	79.4
	Postgraduate	41	11.6
Employer Type	Saudi Red Crescent Authority	134	37.9
	Government Health Sector	129	36.4
	Privet Health Sector	91	25.7
Employer Region	Central region	170	48.0
	Northern region	19	5.4
	Southern region	27	7.6
	Eastern region	71	20.1
	Western region	67	18.9
Experience	<1 year	96	27.1
	1 to 5 years	166	46.9
	6 to 10 years	48	13.6
	11 to 15 years	28	7.9
	16 years and above	16	4.5

**Table 2:** Descriptive statistics and *t*-test for elements for manager behavior.

Sr.	Elements My Supervisor…	Mean	Std. Deviation	Coefficient of Variation	*t*-Test	Sig.
1	… pays attention to my feelings and problems and notices if I’m not feeling so well	2.70	1.277	0.47	−4.369	0.000
2	… shows that they appreciate the way I do my job	2.94	1.331	0.45	−0.838	0.402
3	… helps me with a certain task if necessary	3.01	1.285	0.43	0.124	0.901
4	… gives me advice on how to handle things if necessary	3.02	1.306	0.43	0.244	0.807
5	… would be someone I would speak to if I was experiencing workplace stress	2.61	1.384	0.53	−5.262	0.000
6	… is considerate when managing team members	2.94	1.247	0.42	−0.938	0.349
7	… involves me in decision-making	2.82	1.319	0.47	−2.578	0.10
8	… is accessible and approachable to people in the team	3.17	1.225	0.39	2.603	0.10
9	… remains objective when an issue between staff members arise	3.08	1.265	0.41	1.134	0.257
Manager behavior scale	2.92	1.124	0.38	−1.33	0.184	

**Table 3:** Descriptive statistics and *t*-test for elements for mental well-being.

Sr.	Elements	Mean	Std. Deviation	Coefficient of Variation	*t*-Test	Sig.
1	I’ve been feeling optimistic about the future	3.36	1.083	0.32	6.284	0.000
2	I’ve been feeling useful	3.62	1.156	0.32	10.071	0.000
3	I’ve been feeling relaxed	2.84	1.183	0.42	−2.561	0.011
4	I’ve been dealing with problems well	3.55	1.034	0.29	10.074	0.000
5	I’ve been thinking clearly	3.49	1.060	0.30	8.626	0.000
6	I’ve been feeling close to other people	3.35	1.174	0.35	5.570	0.000
7	I’ve been able to make up my own mind about things	3.58	1.051	0.29	10.415	0.000

**Table 4:** Goodness-of-fit of the used model.

Goodness-of-Fit			
	Value	df	Value/df
Deviance	155.023	318	0.487
Scaled Deviance	354.000	318	
Pearson Chi-Square	155.023	318	0.487
Scaled Pearson Chi-Square	354.000	318	
Log-Likelihood	−356.151		
Akaike’s Information Criterion (AIC)	786.302		
Finite Sample Corrected AIC (AICC)	795.200		
Bayesian Information Criterion (BIC)	929.466		
Consistent AIC (CAIC)	966.466		

**Table 5:** Significance test of the model.

Omnibus Test		
Likelihood Ratio Chi-Square	df	Sig.
152.460	35	0.000

**Table 6:** Parameter estimates for the model.

Parameter	B	Std. Error	Wald Chi-Square	df	Sig.	R-Square		
(Intercept)		2.520		1.4517	3.013	1	0.083	
Sex		-		-	4.431	1	0.035	
Age in Years		-		-	3.215	5	0.667	0.350
Nationality	-	-	0.223	1	0.637			
Residence Area	-	-	49.500	12	0.000			
Marital Status	-	-	0.319	2	0.852			
Level of Education		-		-	4.015	3	0.260	
Employer Type	-	-	3.413	2	0.182			
Employer Region	-	-	6.708	4	0.152			
Employee Experience	-	-	10.243	4	0.037			
Manager Behavior		0.320		0.0350	83.683	1	0.000	

## Data Availability

The datasets used and/or analyzed during the current study are available from the corresponding author uon reasonable request.

## Appendix A

**Table A1:** Frequency distribution and percentage for items of manager behavior.

Sr	Items My Supervisor…	Valid	Never/Hardly Ever	Rarely	Sometimes	Often	Always
1	… pays attention to my feelings and problems and notices if I’m not feeling so well.	n	70	102	89	49	44
		%	19.8	28.8	25.1	13.8	12.4
2	… shows that they appreciate the way I do my job	n	64	77	84	74	55
		%	18.1	21.8	23.7	20.9	15.5
3	… helps me with a certain task if necessary	n	50	79	104	60	61
		%	14.1	22.3	29.4	16.9	17.2
4	… gives me advice on how to handle things if necessary	n	52	79	97	63	63
		%	14.7	22.3	27.4	17.8	17.8
5	… would be someone I would speak to if I was experiencing workplace stress.	n	100	82	78	43	51
		%	28.2	23.2	22	12.1	14.4
6	… is considerate when managing team members	n	53	80	104	70	47
		%	15	22.6	29.4	19.8	13.3
7	…involves me in decision making	n	75	70	103	56	50
		%	21.2	19.8	29.1	15.8	14.1
8	… is accessible and approachable to people in the team	n	31	81	105	71	66
		%	8.8	22.9	29.7	20.1	18.6
9	… remains objective when an issue between staff members arise	n	46	69	114	62	63
		%	13.0	19.5	32.2	17.5	17.8
Total		n	541	719	878	548	500
		%	16.98	22.57	32.2	17.5	17.8

## Appendix B

**Table A2:** Frequency distribution and percentage for items of mental well-being dimension.

Sr.	Items	Valid	None of the Time	Rarely	Some of the Time	Often	All of the Time
1	I’ve been feeling optimistic about the future	n	21	44	134	96	59
		%	5.9	12.4	37.9	27.1	16.7
2	I’ve been feeling useful.	n	22	36	89	115	92
		%	6.2	10.2	25.1	32.5	26.0
3	I’ve been feeling relaxed.	n	49	96	109	63	37
		%	13.8	27.1	30.8	17.8	10.5
4	I’ve been dealing with problems well.	n	17	32	105	138	62
		%	4.8	9	29.7	39	17.5
5	I’ve been thinking clearly	n	18	41	105	131	59
		%	5.1	11.6	29.7	37	16.7
6	I’ve been feeling close to other people	n	25	65	89	112	63
		%	7.1	18.4	25.1	31.6	17.8
7	I’ve been able to make up my own mind about things	n	15	37	99	133	70
		%	4.2	10.5	28	37.6	19.8
Total		n	167	351	730	788	442
		%	6.74	14.16	29.46	31.80	17.84

## Appendix C

**Table A3:** The variance analysis of the study’s dimensions according to gender.

Dimensions	Gender	N	Mean Rank	Mann–Whitney	Sig.
Manager Behavior	Male	299	171.90	6547.000	0.016
	Female	55	207.96		
Mental Well-Being	Male	299	179.51	7620.500	0.387
	Female	55	166.55		

## Appendix D

**Table A4:** The variance analysis of the study’s dimensions according to age.

Dimensions	Age	N	Mean Rank	Kruskal–Wallis	Sig.
Manager Behavior	<25	68	216.63	17.22	0.004
	25–34 years	239	167.25		
	35–44 years	37	175.11		
	45–54 years	6	217.75		
	55–60 years	3	112.00		
	>60	1	10.00		
Mental Well-Being	<25	68	190.07	8.710	0.121
	25–34 years	239	173.38		
	35–44 years	37	178.00		
	45–54 years	6	254.42		
	55–60 years	3	118.83		
	>60	1	3.50		

## Appendix E

**Table A5:** The variance analysis of the study’s dimensions according to residence.

Dimensions	Residence	N	Mean Rank	Kruskal–Wallis	Sig.
Manager Behavior	Riyadh Region	178	171.52	26.863	0.008
	Makkah Region	60	198.43		
	Madinah Region	15	160.90		
	Qasim Region	6	145.92		
	Eastern Province	58	204.54		
	Asir Region	8	173.13		
	Tabuk Region	6	65.33		
	Hail Region	3	192.83		
	Jazan Region	5	250.60		
	Najran Region	9	126.06		
	Bahah Region	1	10.00		
	Outside Saudi Arabia	2	44.75		
	Unknown	3	134.33		
Mental Well-Being	Riyadh Region	178	166.39	3.185	0.000
	Makkah Region	60	202.15		
	Madinah Region	15	99.57		
	Qasim Region	6	167.33		
	Eastern Province	58	209.79		
	Asir Region	8	167.44		
	Tabuk Region	6	135.00		
	Hail Region	3	243.50		
	Jazan Region	5	251.50		
	Najran Region	9	123.28		
	Bahah Region	1	349.50		
	Outside Saudi Arabia	2	10.75		
	Unknown	3	268.33		

## Appendix F

**Table A6:** The variance analysis of the study’s dimensions according to years of experience.

Dimensions	Years of Experience	N	Mean Rank	Kruskal–Wallis	Sig.
Manager Behavior	<1 year	96	213.88	18.725	0.001
	1 to 5 years	166	167.03		
	6 to 10 years	48	153.22		
	11 to 15 years	28	177.80		
	16 years and above	16	140.19		
Mental Well-Being	<1 year	96	187.73	5.151	0.272
	1 to 5 years	166	166.01		
	6 to 10 years	48	176.51		
	11 to 15 years	28	197.89		
	16 years and above	16	202.63		
